# Factors associated with the transformation of intention into action in influenza vaccination among healthcare professionals: a cross-sectional study

**DOI:** 10.3389/fpubh.2026.1926271

**Published:** 2026-08-17

**Authors:** Qin Tang, Ruixue Hu, Mei Cha, Qin Zhang, Jinyan Chen, Yuan Yuan

**Affiliations:** 1 Vaccine Clinic, Chengdu Women’s and Children’s Central Hospital, School of Medicine, University of Electronic Science and Technology of China, Chengdu, China; 2 Department of Sterile Processing Nursing, West China Second University Hospital, Sichuan University, Chengdu, China; 3 Key Laboratory of Birth Defects and Related Diseases of Women and Children (Sichuan University), Ministry of Education, Chengdu, China; 4 Department of Public Health, Chengdu Women’s and Children’s Central Hospital, School of Medicine, University of Electronic Science and Technology of China, Chengdu, China

**Keywords:** factors, healthcare professionals, influenza vaccine, vaccination action, vaccination intention

## Abstract

**Introduction:**

This study aimed to investigate the intention-action discrepancy in influenza vaccination and identify factors associated with vaccination behavior among healthcare professionals.

**Methods:**

This was a cross-sectional study. An anonymous questionnaire survey was conducted with healthcare professionals at a hospital in Chengdu, China from May to June 2025. Stratified convenience sampling was used. Data concerning respondents’ sociodemographic characteristics, knowledge of influenza and vaccines, and perceptions of and attitudes toward vaccination were collected. The chi-square test was performed for comparisons between intentions and actions. Multivariate logistic regression analysis was performed to analyze the factors associated with healthcare professionals’ vaccination behavior.

**Results:**

A total of 570 questionnaires were distributed, and 547 (95.95%) valid responses were collected. Among the 547 healthcare professionals, 316 (57.77%) intended to be vaccinated, and 251 (45.89%) were vaccinated during the previous influenza season. Having the intention to be vaccinated but not being vaccinated during the previous influenza season was considered an intention-action discrepancy. Among the 316 healthcare professionals who intended to be vaccinated, 96 were not vaccinated, indicating an intention-action discrepancy rate of 30.38%. Healthcare professionals who intended to be vaccinated but were not vaccinated accounted for 17.55% (96/547) of the participants finally included in the study. Professional title (odds ratio (OR) = 1.421, 95% confidence interval (CI): 1.079–1.872), hospital department type (OR = 0.467, 95% CI: 0.312–0.698), mastery of knowledge about influenza and vaccines (OR = 1.874, 95% CI: 1.256–2.807), concerns about severe adverse reactions following vaccination (OR = 2.479, 95% CI: 1.646–3.733), opinion on the statement that receiving an influenza vaccine is an effective way to reduce the incidence of severe influenza (OR = 0.427, 95% CI: 0.214–0.852), and awareness of the hospital’s free or convenient vaccination services (OR = 0.217, 95% CI: 0.126–0.374) were major factors associated with healthcare professionals’ vaccination behavior (all *p* < 0.05).

**Conclusion:**

A **s**trong intention to be vaccinated and a low vaccination rate were observed among healthcare professionals, indicating an intention-action discrepancy. It is advisable to strengthen targeted health education and optimize vaccination services to promote the transformation of intention into action in influenza vaccination among healthcare professionals.

## 1 Introduction

Influenza is an acute respiratory infectious disease caused by influenza viruses. It is characterized by rapid spread, strong infectivity, and widespread susceptibility among the population. Symptoms such as fever and cough may occur in people with influenza. In severe cases, complications such as pneumonia and respiratory failure may occur, posing a threat to patients’ lives (1, 2). Healthcare professionals often come into direct contact with various patients and have a significantly higher probability of being infected with influenza viruses than the general population. Influenza virus infection among healthcare professionals may affect the implementation of healthcare work and cause influenza viruses to spread within hospitals, thereby posing a serious threat to patients, colleagues, and hospital operations (3, 4). Vaccination is an effective means of preventing influenza and reducing the incidence of severe cases and mortality caused by influenza (5). Therefore, increasing vaccination rates among healthcare professionals is a key measure to reduce the risk of nosocomial influenza transmission, ensure healthcare safety, and promote public health.

China has been promoting influenza vaccination consistently over the past few years. The Chinese government has released policies to promote vaccination among healthcare professionals, but vaccination coverage has not reached the desired level (6). Previous studies have identified an inconsistency between strong intentions to get vaccinated and low vaccination rates among some healthcare professionals (7, 8). Whether this is a common problem among healthcare professionals remains unclear. Understanding the intention-action gap in receiving the influenza vaccine among healthcare professionals and identifying the associated factors are critical for formulating targeted intervention strategies and increasing vaccination coverage. This study used a questionnaire to survey healthcare professionals at a hospital in China from May to June 2025. It aimed to clarify the discrepancy between the intention to receive an influenza vaccine and the act of doing so and to identify factors associated with vaccination behavior. This study may provide evidence for optimizing strategies to promote influenza vaccination among healthcare professionals and strengthening infection prevention and control in hospitals, thereby ensuring smooth healthcare operations.

## 2 Materials and methods

### 2.1 Ethics approval and consent to participate

This study was conducted in accordance with the Declaration of Helsinki. All research methods were carried out in accordance with the relevant guidelines and regulations. Ethics approval of this study was obtained from the Ethics Committee of Chengdu Women’s and Children’s Central Hospital [Ethical Approval for Scientific Research 2025 (21)-2]. Written informed consent to participate was obtained from all of the participants in the study.

### 2.2 Study participants

This study was conducted among healthcare professionals at a tertiary Grade A hospital in Chengdu, China. Stratified sampling was performed according to hospital department type: clinical, para-clinical, and administrative and logistics departments. According to the Guideline on Influenza Vaccination for Staff in Chinese Medical Institutions (9), clinical departments were divided into high-risk departments (Emergency Department, Fever Clinic, Department of Respiratory Medicine, Department of Infectious Diseases, and Intensive Care Unit) and non-high-risk clinical departments. The inclusion of healthcare professionals from different departments and with varying professional titles and years of work ensured the representativeness of the survey participants. A total of 570 healthcare professionals were selected to participate in the study.

The inclusion criteria were as follows: ① healthcare professionals who were regular employees of the surveyed hospital, including doctors, nurses, paramedical personnel, such as staff working in the Department of Laboratory Medicine, Department of Radiology, and Department of Ultrasound, and administrative and logistical personnel; and ② individuals who provided informed consent and voluntarily participated in this questionnaire survey.

The exclusion criteria were as follows: ① visiting staff, interns, or personnel receiving the hospital’s standardized training; ② individuals with medical contraindications to receiving the influenza vaccine, such as a history of severe allergies or a weak immune system, who provided relevant medical evidence; or ③ individuals who refused to participate or could not submit valid responses to the questionnaire survey.

### 2.3 Survey tools

The researchers drafted the Questionnaire on Influenza Vaccination Among Healthcare Professionals according to the study purpose and literature published in China and abroad (10, 11). Six experts were invited to provide their opinions on the drafted questionnaire. A final questionnaire was then formulated through expert consensus. The six experts who participated in the expert consultation had more than 10 years of relevant work experience. Among them, two had senior professional titles, two had associate senior professional titles, and two had mid-level professional titles; four had master’s degrees, and two had bachelor’s degrees.

A survey pretest was conducted with 30 healthcare professionals from a tertiary Grade A hospital in Chengdu, China. The time required to complete the questionnaire was 10 to 15 min. Problems encountered during pretesting were promptly resolved before the survey. The Cronbach’s alpha coefficient for the questionnaire was 0.82, and the content validity index was 0.91, indicating good reliability and validity.

The questionnaire consisted of four parts: ① sociodemographic characteristics, including gender, age, educational attainment, professional title, length of work experience, and type of department in which the respondent worked; ② knowledge of influenza and vaccines, including the transmission routes of influenza viruses, signs and symptoms, danger of influenza, target populations for influenza vaccination, timing of vaccination, vaccine efficacy, and contraindications. This part consisted of 10 items. Each item was scored 1 point for a correct answer and 0 points if the respondent gave an incorrect response or chose the response option “I do not know.” The total score for this part ranged from 0 to 10. A score of ≥ 6 indicated that the respondent had mastered knowledge of influenza and vaccines, while a score of < 6 indicated that the respondent lacked such mastery; ③ intentions and actions regarding influenza vaccination; and ④ factors associated with, and reasons for, taking action to get vaccinated, including respondents’ perception of the danger of influenza, perception of the safety and effectiveness of influenza vaccines, and perception of vaccination convenience.

This anonymous cross-sectional survey was conducted from May to June 2025. The investigators received standardized training before the survey to understand the questionnaire instructions and key considerations. The questionnaire was generated using Wenjuanxing, a popular Chinese questionnaire survey platform. The questionnaire link and QR code were shared with all healthcare professionals in the WeChat group chat of the surveyed hospital to invite them to participate in the survey. The questionnaire contained standardized instructions explaining the purpose of the survey and the relevant definitions. All questions were mandatory. Responses could be submitted only after all questions had been answered. Excel data were exported from the Wenjuanxing platform after the survey. Two researchers then reviewed and summarized the data and screened the data using the platform’s backend. Responses with a survey completion time of ≤ 30 s and responses that lacked logical coherence were removed. A total of 570 questionnaires were distributed, and 547 valid responses were collected, with an effective response rate of 95.96%.

### 2.4 Statistical analysis

SPSS 25.0 was used for data analysis. Enumeration data were expressed as frequencies (*n*) and percentages (%), and the chi-square (*χ*^2^) test was performed for comparisons between groups. Measurement data were expressed as mean ± standard deviation ( $\bar{x}\pm s$ ), and comparisons between groups were conducted using a *t*-test or analysis of variance. Receipt of vaccination (yes = 1, no = 0) was treated as the dependent variable, and variables with *p* < 0.05 in the univariate analysis were considered independent variables. These variables were included in the multivariate logistic regression model to analyze the key factors associated with healthcare professionals’ vaccination behavior. A statistically significant difference was identified by *p* < 0.05.

## 3 Results

### 3.1 Sociodemographic characteristics of the study participants

Among the 547 healthcare professionals finally included in the study, 503 (91.96%) were females and 44 (8.04%) were males; 326 (59.60%) were aged between 30 and 44 years; 341 (62.34%) held a bachelor’s degree; 436 (79.71%) had mid-level, junior or lower professional titles; 401 (73.31%) had work experience of ≤ 20 years; 324 (59.23%) were from high-risk departments, and 223 (40.77%) were from non-high-risk departments, including non-high-risk clinical departments, para-clinical departments, and administrative and logistics departments, as shown in Table 1.

**Table 1:** Sociodemographic characteristics of the study participants.

Characteristics	Frequency (*n*)	Percentage (%)
Gender		
Female	503	91.96
Male	44	8.04
Age (years)		
<30	90	16.45
30–44	326	59.60
≥45	131	23.95
Educational attainment		
Associate degree or below	78	14.26
Bachelor’s degree	341	62.34
Master’s degree or higher	128	23.40
Professional title		
Junior or below	177	32.36
Mid-level	259	47.35
Senior	111	20.29
Years of work experience		
<10	195	35.65
10–20	206	37.66
>20	146	26.69
Hospital department type		
High-risk departments	324	59.23
Non-high-risk departments	223	40.77

### 3.2 Characteristics of intention-action discrepancy in influenza vaccination

This study used survey data from 547 healthcare professionals and focused on the discrepancy between their intention to receive an influenza vaccine and their subsequent actions. This study examined the obstacles to the transformation of intention into action among healthcare professionals (Table 2). Among the 547 healthcare professionals, 316 (57.77%) intended to receive an influenza vaccine, and 251 (45.89%) received one during the previous influenza season. In this study, having the intention to receive an influenza vaccine but not being vaccinated during the previous influenza season was considered an intention-action discrepancy. Among the 316 healthcare professionals who intended to be vaccinated, 220 were vaccinated, giving an intention-action conversion rate of 69.62%, while 96 were not vaccinated, giving an intention-action discrepancy rate of 30.38%. Healthcare professionals who intended to be vaccinated but did not receive vaccination accounted for 17.55% (96/547) of the participants finally included in the study. Among the 231 healthcare professionals who did not intend to be vaccinated, 31 (13.42%) were vaccinated, suggesting that vaccination behavior may not always correspond with stated intentions. This indicated that vaccination intention was not the sole factor associated with vaccination behavior and that an intermediate blocking mechanism existed between intention and action.

**Table 2:** Intention-action discrepancy in influenza vaccination among healthcare professionals.

Vaccination intention	Receipt of vaccination (yes) (*n*)	Receipt of vaccination (no) (*n*)	Total	Intention-action conversion rates/Vaccination rates (%)
Yes	220	96	316	69.62
No	31	200	231	13.42
Total	251	296	547	45.89

### 3.3 Univariate analysis of intention-action discrepancy in influenza vaccination

The chi-square test showed differences in intention-action discrepancy in influenza vaccination among healthcare professionals with different demographic and job characteristics and cognitions (Table 3). Regarding job characteristics, healthcare professionals from high-risk departments and those with senior professional titles had stronger intentions to be vaccinated and higher vaccination rates than those from non-high-risk departments and those with mid-level, junior or lower professional titles. The differences between the groups were statistically significant (all *p* < 0.05). This suggests that the risk of occupational exposure, years of work experience and the accumulation of knowledge about influenza and vaccines may positively influence healthcare professionals’ vaccination behavior. In terms of cognitions, a stronger intention to be vaccinated (*χ*^2^ = 25.187, *p* < 0.05) and a higher vaccination rate (*χ*^2^ = 29.114, *p* < 0.05) were observed among healthcare professionals who had mastered knowledge of influenza and vaccines compared with those who lacked such mastery. This suggests that the accumulation of knowledge about influenza and vaccines is a significant factor associated with healthcare professionals’ decision to get vaccinated. Cognitive deficiency is a prerequisite that restricts vaccination behavior.

**Table 3:** Intentions and actions regarding influenza vaccination among healthcare professionals with different characteristics.

Characteristics	Total	The number of healthcare professionals who intended to be vaccinated	*χ* ^2^	*p*	The number of healthcare professionals who were vaccinated	*χ* ^2^	*p*
Gender							
Female	503	288	0.675	0.411	230	0.065	0.798
Male	44	28			21		
Age (years)							
<30	90	49	0.592	0.744	32	5.307	0.070
30–44	326	192			150		
≥45	131	75			67		
Educational attainment							
Associate degree or below	78	38	5.293	0.071	32	2.545	0.280
Bachelor’s degree	341	195			153		
Master’s degree or higher	128	83			66		
Professional title							
Junior or below	177	88	7.311	0.026	64	13.208	0.001
Mid-level	259	157			123		
Senior	111	71			64		
Years of work experience							
<10	195	116	0.367	0.833	80	3.086	0.214
10–20	206	117			98		
>20	146	83			73		
Hospital department type							
High-risk departments	324	204	8.786	0.003	168	11.389	0.001
Non-high-risk departments	223	112			83		
Knowledge assessment result							
<6 points	158	65	25.187	<0.001	44	29.114	<0.001
≥6 points	389	251			207		

### 3.4 Univariate analysis of perceptions of and attitudes toward vaccination

The questionnaire survey on healthcare professionals’ perceptions of and attitudes toward vaccination showed that the factors associated with healthcare professionals’ actions regarding vaccination included their perceptions of the danger of influenza, perceptions of the safety and effectiveness of influenza vaccines, their awareness of influenza vaccine updates, and their awareness of the hospital’s free or convenient vaccination services (all *p* < 0.05), as shown in Table 4. This suggests that healthcare professionals’ vaccination behavior is influenced not only by their level of knowledge about influenza and vaccines but also by their health beliefs and the hospital’s vaccination services.

**Table 4:** The influences of healthcare professionals’ perceptions of and attitudes toward vaccination on their vaccination behavior.

Associated factors	Responses	The number of respondents	The number of respondents who were vaccinated	*χ* ^2^	*p*
Influenza is an acute respiratory infectious disease that poses a serious threat to human health	Agree	419	199	1.863	0.172
	Disagree	128	52		
Healthcare professionals have a greater risk of being infected with influenza viruses compared with the general population	Agree	494	238	10.782	0.001
	Disagree	53	13		
Receiving an influenza vaccine is an effective measure for preventing influenza	Agree	403	224	57.965	<0.001
	Disagree	144	27		
Receiving an influenza vaccine is an effective way to reduce the incidence of severe influenza	Agree	426	231	53.928	<0.001
	Disagree	121	20		
Concerns about experiencing severe adverse reactions following influenza vaccination	Yes	211	71	20.716	<0.001
	No	336	180		
Awareness of the 2024 China Influenza Vaccination Guidelines issued by the Chinese Center for Disease Control and Prevention	Yes	257	124	1.090	0.297
	No	290	127		
Awareness of influenza vaccine updates	Yes	389	190	4.741	0.029
	No	158	61		
Awareness of the hospital’s free or convenient vaccination services	Yes	352	216	95.253	<0.001
	No	195	35		

### 3.5 Multivariate logistic regression analysis of action to get vaccinated

Receipt of vaccination was treated as the dependent variable (yes = 1, no = 0), and variables with *p* < 0.05 in the univariate analysis, including professional title, department type, knowledge assessment result, and perceptions of and attitudes toward vaccination, were considered independent variables and included in the multivariate logistic regression model. In terms of job characteristics, healthcare professionals with higher professional titles and healthcare professionals from high-risk department were more likely to be vaccinated, compared with those with lower professional titles (odds ratio (OR) = 1.421, *p* < 0.05) and from non-high-risk departments (OR = 0.467, *p* < 0.05). This suggests that a high risk of occupational exposure and extensive work experience can significantly increase healthcare professionals’ willingness to be vaccinated. In terms of cognitions, healthcare professionals who had mastered knowledge of influenza and vaccines (OR = 1.874, *p* < 0.05), agreed with the statement that receiving an influenza vaccine is an effective way to reduce the incidence of severe influenza (OR = 0.427, *p* < 0.05), and had no concerns about severe adverse reactions following vaccination (OR = 2.479, *p* < 0.05) were more likely to be vaccinated. This indicates that positive health beliefs and adequate knowledge are the principal internal factors driving the transformation of intention into action. In terms of hospital services, healthcare professionals who were aware of the hospital’s free or convenient vaccination services were more likely to be vaccinated than those who were not aware of such services (OR = 0.217, *p* < 0.001) (Table 5). This indicates that the provision of free or convenient vaccination services by hospitals is a key external controllable factor associated with healthcare professionals’ vaccination behavior. This suggests that optimizing the services of healthcare institutions is an important intervention measure to overcome the obstacle to the transformation of intention into action and increase the overall vaccination rate.

**Table 5:** Multivariate logistic regression analysis of factors associated with the transformation of intention into action in influenza vaccination.

Grouping	*β*	SE	Wald *χ*^2^	*p*	Odds ratio	95% confidence interval
Professional title (Junior or below = 1, mid-level = 2, senior = 3)	0.352	0.140	6.265	0.012	1.421	1.079–1.872
Hospital department type (high-risk departments = 1, non-high-risk departments = 2)	−0.762	0.206	13.715	<0.001	0.467	0.312–0.698
Mastery of knowledge about influenza and vaccines (no = 1, yes = 2)	0.626	0.214	8.652	0.003	1.874	1.256–2.807
Healthcare professionals have a greater risk of being infected with influenza viruses compared with the general population (agree = 1, disagree = 2)	−0.208	0.392	0.283	0.595	0.812	0.377–1.749
Receiving an influenza vaccine is an effective measure for preventing influenza (agree = 1, disagree = 2)	−0.103	0.347	0.088	0.766	0.902	0.457–1.782
Receiving an influenza vaccine is an effective way to reduce the incidence of severe influenza (agree = 1, disagree = 2)	−0.850	0.352	5.826	0.016	0.427	0.214–0.852
Concerns about experiencing severe adverse reactions following influenza vaccination (yes = 1, no = 2)	0.908	0.209	18.866	<0.001	2.479	1.646–3.733
Awareness of influenza vaccine updates (yes = 1, no = 2)	−0.204	0.226	0.812	0.367	0.816	0.523–1.271
Awareness of the hospital’s free or convenient vaccination services (yes = 1, no = 2)	−1.528	0.277	30.348	<0.001	0.217	0.126–0.374

## 4 Discussion

This study showed that 57.77% of healthcare professionals intended to receive an influenza vaccine, and the vaccination rate during the previous influenza season was 45.89%, indicating an intention-action discrepancy in vaccination. This is consistent with the results of some studies conducted in China (12, 13). Among the 316 healthcare professionals who intended to be vaccinated, 96 were not vaccinated. Having the intention to be vaccinated but not being vaccinated during the previous influenza season was considered an intention-action discrepancy in this study. The intention-action discrepancy is a common obstacle hindering the establishment of an immunity barrier among healthcare professionals. Internal psychological barriers and external conditions are the principal mediating factors associated with the intention-action discrepancy (14). From the perspective of the Health Belief Model, the formation of intention is driven by the perception of disease susceptibility and the cognition of vaccination benefits, while behavior is additionally constrained by perceived barriers, action convenience, and external organizational support, which are controllable external conditions (15). The differences in the factors driving intention and action may explain why 30.38% of healthcare professionals with an intention to be vaccinated did not get vaccinated in this study. Therefore, subsequent interventions should extend beyond routine health education. More attention should be paid to healthcare professionals who intend to be vaccinated but are not vaccinated, to eliminate the specific factors that hinder the transformation of intention into action.

Multivariate logistic regression analysis revealed that professional title and department type were key factors associated with action regarding vaccination. Healthcare professionals with senior professional titles had a significantly higher vaccination rate than those with lower professional titles. This may be due to their higher level of awareness of occupational risks, richer clinical experience, and stronger sense of responsibility for health. After other factors were controlled for, healthcare professionals from non-high-risk departments were less likely to be vaccinated than those from high-risk departments. In this study, the assignment for department type was as follows: high-risk departments = 1 and non-high-risk departments = 2. An OR value of 0.467 was identified, indicating that healthcare professionals from non-high-risk departments had a lower probability of vaccination than those from high-risk departments. This suggests that perceptions of infection risk remain an important factor associated with healthcare professionals’ vaccination behavior. Healthcare professionals from high-risk departments had a higher probability of direct contact with patients with confirmed or suspected influenza, so they had a deeper understanding of the risk of infection. Therefore, they had a stronger intention to be vaccinated and a higher vaccination rate than those from non-high-risk departments. This is consistent with the results of Wang et al. (16) and Tao et al. (17). Healthcare professionals from non-high-risk departments had lower vaccinate rates, which may be due to lower perceptions of infection risk and insufficient attention to vaccination. Therefore, hospitals need to provide targeted publicity and training for healthcare professionals (18). Training on the hazards of hospital-acquired influenza infections and the safety of vaccines is recommended for healthcare professionals with low professional titles. This training could help alleviate concerns about adverse reactions following vaccination. Training on the risk of occupational exposure is recommended for healthcare professionals from non-high-risk departments to enhance their awareness of protection against occupational exposure.

An adequate understanding of influenza and vaccines is also an important factor associated with healthcare professionals’ vaccination behavior. Healthcare professionals who had mastered knowledge of influenza and vaccines had a significantly higher vaccination rate than those who lacked such mastery. This indicates that an adequate understanding of the danger of influenza, the protective efficacy of vaccines, and the necessity of vaccination could improve the intention–action conversion rate for vaccination among healthcare professionals (19–21). In this study, 28.88% of healthcare professionals lacked mastery of knowledge about influenza and vaccines, indicating inadequacy in health education they had received. Targeted health education needs to be strengthened, especially for those who lacked mastery of the relevant knowledge. A combination of various approaches, including in-person training, brochures and the online dissemination of health information, is recommended for improving knowledge of influenza and vaccines.

In this study, healthcare professionals’ perceptions of the safety and effectiveness of influenza vaccines was the primary factor associated with their vaccination actions. This suggests that eliminating cognitive misunderstandings is key to improving vaccination rates. Concerns about side effects are a common factor associated with vaccination coverage worldwide (22, 23). In this study, yes = 1 and no = 2 were used for assignment of the variable “concerns about severe adverse reactions following influenza vaccination,” and an OR value of 2.479 was observed, indicating that healthcare professionals who were not concerned about severe adverse reactions were more likely to be vaccinated than those who were concerned. Therefore, concerns about vaccine safety remain an important factor hindering healthcare professionals’ vaccination. Some healthcare professionals may have an inadequate understanding of adverse reactions, such as fever, fatigue and local pain following vaccination, or may be excessively concerned about the risk of severe adverse reactions, thereby affecting their vaccination decisions. Strengthening the promotion of evidence on the safety and effectiveness of influenza vaccines is recommended in subsequent interventions to reduce unnecessary risk-related concerns.

Healthcare professionals’ awareness of free or convenient vaccination services is also an important factor associated with their decision to be vaccinated. In this study, yes = 1 and no = 2 were assigned to the variable “awareness of the hospital’s free or convenient vaccination services,” and an OR value of 0.217 was observed, indicating that those who were not aware of the hospital’s free and convenient vaccination services were significantly less likely to be vaccinated. Considering that the study subjects came from the same hospital, this variable is more appropriately interpreted as healthcare professionals’ awareness of the surveyed hospital’s free or convenient vaccination services, rather than differences in services across hospitals. This result suggests that even when a hospital provides vaccination services, it must improve healthcare professionals’ awareness of and access to them through clear notifications, a designated vaccination location, extended service hours, departmental appointments or on-site vaccination services. This result confirms that the accessibility and affordability of vaccination services are important external factors associated with healthcare professionals’ vaccination behavior (24, 25). Although the provision of free influenza vaccination services for healthcare professionals has been advocated in some regions of China, some healthcare institutions in other regions have not implemented it. Complicated vaccination procedures and inflexible vaccination service hours can result in insufficient convenience for healthcare professionals seeking vaccination. Therefore, optimizing vaccination services and reducing the time and economic costs of vaccination are effective ways to increase vaccination rates.

This study has several limitations. First, this was a cross-sectional survey, making it impossible to determine causal relationship among the variables. Second, the study sample was drawn from a single hospital, which may introduce regional limitations. The results should therefore be generalized to other regions with caution. Third, factors such as healthcare professionals’ vaccination history and underlying diseases, which might affect their response to vaccination, were not considered. A cohort study with a larger sample size and more potential associated factors can be conducted in the future to validate the study results and explore more effective intervention strategies.

## 5 Conclusion

In this study, the percentage of healthcare professionals who intended to be vaccinated was higher than the vaccination rate. This indicated the presence of barriers to the transformation of intention into action regarding vaccination. Factors such as professional title, department type, knowledge of influenza and vaccines, perceptions of vaccine safety and effectiveness, and convenience of vaccination services are major factors associated with healthcare professionals’ vaccination actions. More attention should be paid to healthcare professionals who intend to be vaccinated but do not receive vaccination, and hospital vaccination services should be optimized to reduce time and economic costs, thereby promoting the transformation of intention into action regarding vaccination. Multiple measures, such as strengthening targeted health education and optimizing vaccination services, can be adopted to increase vaccination rates among healthcare professionals. These measures could help strengthen defenses against nosocomial influenza infection and protect the health and safety of healthcare professionals and patients.

## Data Availability

The raw data supporting the conclusions of this article will be made available by the authors, without undue reservation.
